# Bis{4-bromo-2-[(2-hy­droxy­eth­yl)imino­meth­yl]phenolato-κ^3^ *O*,*N*,*O*′}cadmium

**DOI:** 10.1107/S1600536811025852

**Published:** 2011-07-06

**Authors:** Jing Yu

**Affiliations:** a School of Biological and Chemical Engineering, Jiaxing University, Jiaxing Zhejiang 314001, People’s Republic of China

## Abstract

The centrosymmetric title compound, [Cd(C_9_H_9_BrNO_2_)_2_], was obtained by the reaction of 5-bromo­salicyl­aldehyde, 2-amino­ethanol and cadmium nitrate in ethanol. The Cd atom, located on an inversion centre, is hexa­coordinated by two Schiff base ligands in an octa­hedral coordination through the phenolate O atom, the imine N atom and the hy­droxy O atoms. In the crystal, mol­ecules are linked through inter­molecular O—H⋯O hydrogen bonds, forming chains along the *b* axis.

## Related literature

For the structures and properties of Schiff base Cd complexes, see: Sarkar *et al.* (2011 ▶); Das *et al.* (2010 ▶); Fang & Nie (2010 ▶); Niu *et al.* (2010 ▶); Keypour *et al.* (2009 ▶).
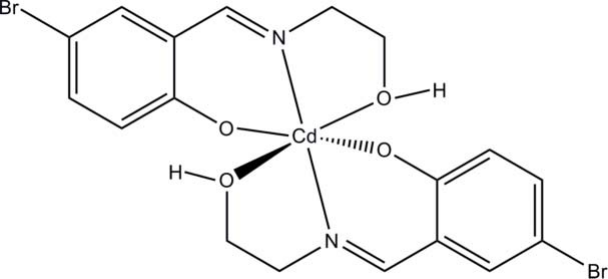


## Experimental

### 

#### Crystal data

[Cd(C_9_H_9_BrNO_2_)_2_]
*M* *_r_* = 598.56
Monoclinic, 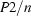

*a* = 10.207 (4) Å
*b* = 5.3275 (19) Å
*c* = 18.656 (7) Å
β = 99.156 (4)°
*V* = 1001.5 (6) Å^3^
*Z* = 2
Mo *K*α radiation
μ = 5.11 mm^−1^
*T* = 298 K
0.23 × 0.20 × 0.20 mm

#### Data collection

Bruker SMART 1K CCD area-detector diffractometer
Absorption correction: multi-scan (*SADABS*; Sheldrick, 2004 ▶) *T*_min_ = 0.386, *T*_max_ = 0.428
7794 measured reflections
2172 independent reflections
1524 reflections with *I* > 2σ(*I*)
*R*_int_ = 0.039

#### Refinement

*R*[*F* ^2^ > 2σ(*F* ^2^)] = 0.055
*wR*(*F* ^2^) = 0.126
*S* = 1.02
2172 reflections
126 parameters
1 restraint
H atoms treated by a mixture of independent and constrained refinement
Δρ_max_ = 1.41 e Å^−3^
Δρ_min_ = −1.55 e Å^−3^

### 

Data collection: *SMART* (Bruker, 2001 ▶); cell refinement: *SAINT* (Bruker, 2001 ▶); data reduction: *SAINT*; program(s) used to solve structure: *SHELXTL* (Sheldrick, 2008 ▶); program(s) used to refine structure: *SHELXTL*; molecular graphics: *SHELXTL*; software used to prepare material for publication: *SHELXTL* and local programs.

## Supplementary Material

Crystal structure: contains datablock(s) I, global. DOI: 10.1107/S1600536811025852/hg5060sup1.cif

Structure factors: contains datablock(s) I. DOI: 10.1107/S1600536811025852/hg5060Isup2.hkl

Additional supplementary materials:  crystallographic information; 3D view; checkCIF report

## Figures and Tables

**Table 1:** Hydrogen-bond geometry (Å, °)

*D*—H⋯*A*	*D*—H	H⋯*A*	*D*⋯*A*	*D*—H⋯*A*
O2—H2⋯O1^i^	0.85 (1)	1.75 (2)	2.599 (7)	173 (9)

Symmetry code: (i) 
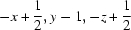
.

**Fig. 1.**
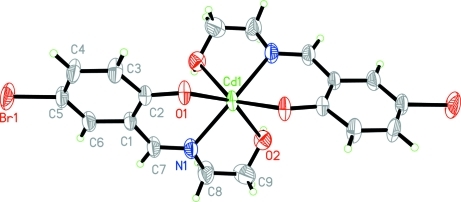
The molecular structure of (I), with atom labels and 30% probability displacement ellipsoids for non-H atoms. Unlabelled atoms are at the symmetry position 1/2 - x, y, 1/2 - z.

**Fig. 2.**
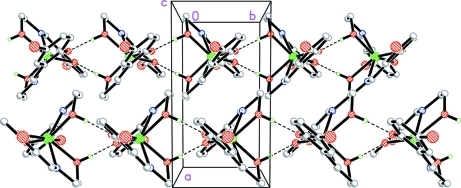
The packing of (I), viewed down the c axis. Hydrogen bonds are drawn as dashed lines. H atoms not involved in hydrogen bonding have been omitted.

## supplementary crystallographic information

### Comment

Schiff base cadmium(II) complexes have been received much attention due to their interesting structures and luminescent properties (Sarkar *et al.*, 2011; Das *et al.*, 2010; Fang & Nie, 2010; Niu *et al.*, 2010; Keypour *et al.*, 2009).

The molecule of the title complex, (I) (Fig. 1), is centrosymmetric, with the inversion center located at the Cd atom. The Cd atom is hexa-coordinated by two Schiff base ligands, forming an octahedral coordination. The Schiff base coordinates to the Co atom through the phenolate O atom, the imine N atom, and the hydroxy O atom. The bond lengths are within normal values. In the crystal, molecules are linked through intermolecular O—H···O hydrogen bonds (Table 1), to form chains along the *b* axis, Fig. 2.

### Experimental

To a solution of 5-bromosalicylaldehyde (0.181 g, 1.0 mmol), 2-aminoethanol (0.061 g, 1.0 mmol) in 20 ml absolute ethanol was added slowly a solution of cadmium nitrate (0.154 g, 0.5 mmol) in ethanol. The mixture was stirred for 2 h at room temperature to give a colorless solution, which was filtered and the filtrate was left to stand at room temperature. Colorless block crystals suitable for X-ray diffraction were obtained by slow evaporation.

### Refinement

H2 atom bonded to O2 atom was located in a difference map and refined with distance restraint of O—H = 0.85 (1) Å. Other H atoms were positioned geometrically and refined using a riding model, with C—H = 0.93–0.97 Å.

### Crystal data

**Table:**

[Cd(C_9_H_9_BrNO_2_)_2_]	*F*(000) = 580
*M**_r_* = 598.56	*D*_x_ = 1.985 Mg m^−^^3^
Monoclinic, *P*2/*n*	Mo *K*α radiation, λ = 0.71073 Å
Hall symbol: -P 2yac	Cell parameters from 1609 reflections
*a* = 10.207 (4) Å	θ = 2.5–24.4°
*b* = 5.3275 (19) Å	µ = 5.11 mm^−^^1^
*c* = 18.656 (7) Å	*T* = 298 K
β = 99.156 (4)°	Block, colorless
*V* = 1001.5 (6) Å^3^	0.23 × 0.20 × 0.20 mm
*Z* = 2	

### Data collection

**Table:**

Bruker SMART 1K CCD area-detector diffractometer	2172 independent reflections
Radiation source: fine-focus sealed tube	1524 reflections with *I* > 2σ(*I*)
graphite	*R*_int_ = 0.039
ω scans	θ_max_ = 27.0°, θ_min_ = 2.1°
Absorption correction: multi-scan (*SADABS*; Sheldrick, 2004)	*h* = −13→12
*T*_min_ = 0.386, *T*_max_ = 0.428	*k* = −6→6
7794 measured reflections	*l* = −23→23

### Refinement

**Table:**

Refinement on *F*^2^	Primary atom site location: structure-invariant direct methods
Least-squares matrix: full	Secondary atom site location: difference Fourier map
*R*[*F*^2^ > 2σ(*F*^2^)] = 0.055	Hydrogen site location: inferred from neighbouring sites
*wR*(*F*^2^) = 0.126	H atoms treated by a mixture of independent and constrained refinement
*S* = 1.02	*w* = 1/[σ^2^(*F*_o_^2^) + (0.034*P*)^2^ + 5.8476*P*] where *P* = (*F*_o_^2^ + 2*F*_c_^2^)/3
2172 reflections	(Δ/σ)_max_ < 0.001
126 parameters	Δρ_max_ = 1.41 e Å^−^^3^
1 restraint	Δρ_min_ = −1.55 e Å^−^^3^

### Special details

**Table:**

Geometry. All e.s.d.'s (except the e.s.d. in the dihedral angle between two l.s. planes) are estimated using the full covariance matrix. The cell e.s.d.'s are taken into account individually in the estimation of e.s.d.'s in distances, angles and torsion angles; correlations between e.s.d.'s in cell parameters are only used when they are defined by crystal symmetry. An approximate (isotropic) treatment of cell e.s.d.'s is used for estimating e.s.d.'s involving l.s. planes.
Refinement. Refinement of *F*^2^ against ALL reflections. The weighted *R*-factor *wR* and goodness of fit *S* are based on *F*^2^, conventional *R*-factors *R* are based on *F*, with *F* set to zero for negative *F*^2^. The threshold expression of *F*^2^ > σ(*F*^2^) is used only for calculating *R*-factors(gt) *etc*. and is not relevant to the choice of reflections for refinement. *R*-factors based on *F*^2^ are statistically about twice as large as those based on *F*, and *R*- factors based on ALL data will be even larger.

### Fractional atomic coordinates and isotropic or equivalent isotropic displacement parameters (Å^2^)

**Table:**

	*x*	*y*	*z*	*U*_iso_*/*U*_eq_	
Cd1	0.2500	0.38621 (12)	0.2500	0.0491 (2)	
Br1	0.21431 (12)	0.4350 (3)	0.64964 (5)	0.1157 (5)	
N1	0.4104 (7)	0.2594 (14)	0.3415 (3)	0.0705 (19)	
O1	0.2194 (5)	0.6681 (8)	0.3344 (2)	0.0577 (13)	
O2	0.3921 (6)	0.0979 (9)	0.2021 (3)	0.0676 (14)	
C1	0.3083 (7)	0.4314 (13)	0.4410 (3)	0.0529 (17)	
C2	0.2245 (7)	0.6144 (12)	0.4037 (3)	0.0476 (15)	
C3	0.1433 (9)	0.7475 (15)	0.4453 (4)	0.073 (2)	
H3	0.0893	0.8751	0.4231	0.087*	
C4	0.1407 (9)	0.6964 (18)	0.5174 (4)	0.081 (3)	
H4	0.0842	0.7849	0.5428	0.097*	
C5	0.2219 (9)	0.5144 (17)	0.5511 (4)	0.069 (2)	
C6	0.3057 (8)	0.3868 (16)	0.5151 (4)	0.067 (2)	
H6	0.3625	0.2677	0.5397	0.080*	
C7	0.4004 (8)	0.2792 (17)	0.4087 (4)	0.073 (2)	
H7	0.4597	0.1840	0.4407	0.088*	
C8	0.5021 (11)	0.066 (2)	0.3211 (5)	0.112 (4)	
H8A	0.5895	0.0913	0.3493	0.134*	
H8B	0.4712	−0.0987	0.3329	0.134*	
C9	0.5113 (10)	0.075 (2)	0.2490 (6)	0.113 (4)	
H9A	0.5551	−0.0772	0.2366	0.136*	
H9B	0.5676	0.2151	0.2409	0.136*	
H2	0.358 (8)	−0.047 (7)	0.194 (5)	0.080*	

### Atomic displacement parameters (Å^2^)

**Table:**

	*U*^11^	*U*^22^	*U*^33^	*U*^12^	*U*^13^	*U*^23^
Cd1	0.0826 (6)	0.0363 (3)	0.0287 (3)	0.000	0.0101 (3)	0.000
Br1	0.1431 (11)	0.1725 (13)	0.0387 (5)	0.0486 (9)	0.0363 (6)	0.0257 (6)
N1	0.082 (5)	0.092 (5)	0.038 (3)	0.029 (4)	0.011 (3)	−0.004 (3)
O1	0.107 (4)	0.038 (2)	0.030 (2)	0.007 (2)	0.018 (2)	0.0017 (18)
O2	0.084 (4)	0.056 (3)	0.064 (3)	−0.005 (3)	0.016 (3)	−0.026 (3)
C1	0.067 (5)	0.060 (4)	0.032 (3)	0.008 (3)	0.008 (3)	0.000 (3)
C2	0.074 (5)	0.037 (3)	0.033 (3)	−0.004 (3)	0.013 (3)	−0.002 (3)
C3	0.114 (7)	0.062 (5)	0.044 (4)	0.024 (5)	0.021 (4)	0.003 (4)
C4	0.111 (7)	0.095 (6)	0.042 (4)	0.033 (6)	0.030 (4)	0.000 (4)
C5	0.089 (6)	0.086 (5)	0.032 (4)	0.017 (5)	0.014 (4)	0.004 (4)
C6	0.083 (6)	0.081 (5)	0.036 (4)	0.021 (5)	0.006 (4)	0.004 (4)
C7	0.088 (6)	0.095 (6)	0.037 (4)	0.035 (5)	0.011 (4)	0.007 (4)
C8	0.112 (8)	0.166 (11)	0.059 (5)	0.078 (8)	0.020 (5)	0.005 (6)
C9	0.084 (7)	0.157 (11)	0.094 (7)	0.031 (7)	0.002 (6)	−0.069 (7)

### Geometric parameters (Å, °)

**Table:**

Cd1—O1	2.233 (4)	C1—C7	1.443 (10)
Cd1—O1^i^	2.233 (4)	C2—C3	1.413 (10)
Cd1—N1	2.272 (6)	C3—C4	1.376 (10)
Cd1—N1^i^	2.272 (6)	C3—H3	0.9300
Cd1—O2^i^	2.382 (5)	C4—C5	1.363 (11)
Cd1—O2	2.382 (5)	C4—H4	0.9300
Br1—C5	1.900 (7)	C5—C6	1.352 (11)
N1—C7	1.278 (8)	C6—H6	0.9300
N1—C8	1.482 (10)	C7—H7	0.9300
O1—C2	1.318 (7)	C8—C9	1.364 (13)
O2—C9	1.387 (11)	C8—H8A	0.9700
O2—H2	0.850 (10)	C8—H8B	0.9700
C1—C2	1.405 (9)	C9—H9A	0.9700
C1—C6	1.407 (9)	C9—H9B	0.9700
			
O1—Cd1—O1^i^	95.5 (2)	C4—C3—C2	122.8 (7)
O1—Cd1—N1	80.5 (2)	C4—C3—H3	118.6
O1^i^—Cd1—N1	124.4 (2)	C2—C3—H3	118.6
O1—Cd1—N1^i^	124.4 (2)	C5—C4—C3	119.1 (7)
O1^i^—Cd1—N1^i^	80.5 (2)	C5—C4—H4	120.4
N1—Cd1—N1^i^	145.4 (4)	C3—C4—H4	120.4
O1—Cd1—O2^i^	90.41 (19)	C6—C5—C4	121.0 (7)
O1^i^—Cd1—O2^i^	149.32 (19)	C6—C5—Br1	119.7 (6)
N1—Cd1—O2^i^	86.3 (2)	C4—C5—Br1	119.3 (6)
N1^i^—Cd1—O2^i^	71.4 (2)	C5—C6—C1	121.1 (7)
O1—Cd1—O2	149.32 (19)	C5—C6—H6	119.5
O1^i^—Cd1—O2	90.41 (19)	C1—C6—H6	119.5
N1—Cd1—O2	71.4 (2)	N1—C7—C1	127.9 (7)
N1^i^—Cd1—O2	86.3 (2)	N1—C7—H7	116.1
O2^i^—Cd1—O2	99.7 (3)	C1—C7—H7	116.1
C7—N1—C8	117.5 (7)	C9—C8—N1	112.1 (8)
C7—N1—Cd1	123.5 (5)	C9—C8—H8A	109.2
C8—N1—Cd1	115.0 (5)	N1—C8—H8A	109.2
C2—O1—Cd1	123.9 (4)	C9—C8—H8B	109.2
C9—O2—Cd1	110.2 (5)	N1—C8—H8B	109.2
C9—O2—H2	109 (6)	H8A—C8—H8B	107.9
Cd1—O2—H2	113 (6)	C8—C9—O2	115.7 (9)
C2—C1—C6	119.8 (6)	C8—C9—H9A	108.3
C2—C1—C7	124.7 (6)	O2—C9—H9A	108.3
C6—C1—C7	115.5 (6)	C8—C9—H9B	108.3
O1—C2—C1	124.2 (6)	O2—C9—H9B	108.3
O1—C2—C3	119.7 (6)	H9A—C9—H9B	107.4
C1—C2—C3	116.2 (6)		

Symmetry codes: (i) −*x*+1/2, *y*, −*z*+1/2.

### Hydrogen-bond geometry (Å, °)

**Table:**

*D*—H···*A*	*D*—H	H···*A*	*D*···*A*	*D*—H···*A*
O2—H2···O1^ii^	0.85 (1)	1.75 (2)	2.599 (7)	173 (9)

Symmetry codes: (ii) −*x*+1/2, *y*−1, −*z*+1/2.
